# Percutaneous Aspiration, Lavage and Antibiotic Instillation

**DOI:** 10.1155/1992/98905

**Published:** 1992

**Authors:** Aws S. Salim

**Affiliations:** Department of Surgery Ward 6 Stobhill General Hospital Glasgow G21 3UW UK


*HPB Surgery*, 1992, Vol. 5, pp. 271–273
Reprints available directly from the publisher
Photocopying permitted by license only

© 1992 Harwood Academic Publishers GmbH
Printed in the United Kingdom

### LETTER TO THE EDITOR

### PERCUTANEOUS ASPIRATION, LAVAGE AND ANTIBIOTIC INSTILLATION

### New Approach in the Management of Acute Cholecystitis Failing to Respond to Conservative Management

Further to my recent publication in the Journal on the percutaneous management of acute calculous cholecystitis[1], I would like to communicate the results of a prospective randomized study conducted at the Medical City in Baghdad, Iraq to examine the role of percutaneous aspiration of the gallbladder followed by lavage and antibiotic instillation into its lumen (500 mg ampicillin with 40 mg gentamicine, PALA) when conservative management of acute cholecystitis appears to be failing.

Patients judged suitable for the study were those presenting with clinical signs and symptoms (including a positive Murphy's sign) of acute cholecystitis coupled with pyrexia (more than 38 °C), tachycardia, leucocytosis, normal serum amylase levels and liver function tests, and ultrasonic evidence of a distended gallbladder containing one or more stones. These patients were treated conservatively. They were completely fasted, confined to bed, given 50 mg pethidine intramuscularly, hydrated intravenously, allowed nil orally for at least 24 hours, and intravenously treated with 1.2 grams of Augmentin (amoxycillin 1 g and clavulanic acid 200 mg, Beecham Research Labs, Brentford, U.K.) every 8 hours. When this treatment proved unsuccessful (failure of fever or tachycardia to show unequivocal evidence of remission or of tenderness and rigidity to subside within 36 hours of beginning nonoperative treatment), patients were fasted, their antibiotic stopped, and after giving written informed consent, they were randomized by drawing sealed envelopes to have a standard cholecystostomy or PALA as previously described[1].

Patients were not recruited if they were demented or moribund on admission, or if they had evidence of septic shock; generalized peritonitis; jaundice; diabetes; pregnancy; alcoholism; sensitivity to penicillins; hepatic, renal or any other serious underlying disease; gastrointestinal disorders like reflux oesophagitis, hiatus herniation, peptic ulceration, the Zollinger-Ellison syndrome, Crohn's disease, ulcerative colitis, or the irritable bowel syndrome; any form of dissolution therapy for cholelithiasis; lithotripsy; percutaneous cholecystolithotomy; previous gastrointestinal surgery; concomitant treatment with any therapy to avoid pharmacological effects of unknown origin; or regular use of analgesics and/or NSAIDs. Furthermore, patients were not studied if ultrasonically the gallbladder was not distended, contained no stones, if there was choledocholithiasis, or dilatation of the biliary tree. At induction of anaesthesia, the cholecystostomy patients received intravenously 2 grams cefotaxime with 500 mg metronidazole. The antibiotics instilled into the gallbladder of the PALA group (500 mg ampicillin and 40 mg gentamicine) were also given intramuscularly in the mentioned doses 8 and 16 hours after the percutaneous procedure. Patients were excluded from efficacy analysis because of failure to comply with the study design or because of using analgesics and/or antispasmodics for abdominal pains. The remaining patients were well matched regarding age, sex ratio, duration of symptoms at presentation (mean in hours: cholecystostomy 11.2 and PALA 10.6) and length of complaints attributed to gallstones (mean in months: cholecystostomy 5.3 and PALA 4.7). In 53 PALA patients (41 women and 12 men, age range 25 to 59 years, mean 36) and 50 cholecystostomy patients (40 women and 10 men, age range 22 to 59 years, mean 35) who were fully evaluable, both procedures were equally effective in controlling the acute attack of cholecystitis within 24 hours, however PALA offered distinctive gains. The procedure incurred no sepsis whereas cholecystostomy was associated with chest infection in 3 patients (6%) and wound infection in 8 patients (16%). PALA avoided an external biliary fistula and enabled a significantly ($p < 0.001$, Mann-Whitney U test) earlier return to free fluids by mouth and then solid food intake relative to cholecystostomy (3 hours vs 50 – 53 hours and 15 – 19 hours vs 56 – 61 hours, respectively). Consequently, the PALA patients were discharged from hospital much sooner ($p < 0.001$) than the cholecystostomy cases (2 days vs 9 days after the intervention). It was noted that the PALA procedure was very well tolerated by all patients and caused no pain or discomfort. None of the patients developed any visible evidence of infection at the site of needle entry and an ultrasound scan carried out 48 hours after the percutaneous intervention showed no evidence of right hypochondrial collection in any case. All the patients in both groups remained asymptomatic until they underwent an elective cholecystectomy 6 weeks later.

Although in many surgical units early cholecystectomy for acute cholecystitis comprises the standard of care and provides optimal therapy[2,3,4], the morbidity and mortality of this procedure particularly in patients who are over 50 years of age, those with jaundice, the obese, and those who have a non-functioning gallbladder, remain higher than those of elective surgery[2,3]. It would, thus, appear that successful conservative management of acute cholecystitis to enable elective cholecystectomy to be carried out at a later date may reduce the overall morbidity and mortality associated with this condition. When the conservative regimen fails to control the acute episode of gallbladder inflammation, cholecystostomy is a simple option not associated with the increased incidence of complications and the higher death rate incurred by cholecystectomy under similar conditions. The acute inflammatory process produces a distended gallbladder with its wall thickened and engorged by active inflammation, which may be surrounded by dense vascular adhesions and the adjacent liver tissue may be congested and friable. In such cases, cholecystectomy carries the risk of haemorrhage which may occur both from separated adhesions and from friable liver tissue. There is also a serious risk of damage to the common bile duct and to the hepatic vessels, if they are obscured by the inflammatory reaction or if there is any lack of experience on the part of the operator. On the other hand, cholecystostomy has limitations in that it is not a definitive procedure and should, therefore, be followed by an elective cholecystectomy. Moreover, it prolongs hospital stay relative to cholecystectomy and produces a higher incidence of wound infection[5]. The biliary fistula of cholecystostomy may take weeks to close and completely heal thereby not only incurring increased nursing demands and additional costs, but also diverting some bile away from the gastrointestinal tract, an event less suitable for normal metabolic activities. The present investigation shows that PALA overcomes the disadvantages associated with cholecystostomy for failure of conservative treatment of acute cholecystitis.

### *References*

1. Salim, A.S. (1991) Percutaneous aspiration, lavage and antibiotic instillation. New approach in the management of acute calculous cholecystitis. *HPB Surgery*, **3**, 167–176
2. Andersson, R., Tranberg, K.-G. and Bengmark, S. (1990) Bile peritonitis in acute cholecystitis. *HPB Surgery*, **2**, 7–13
3. Larmi, T.K.I., Kairaluoma, J.J., Junila, J., Laitinen, S., Ståhlberg, M. and Fock, H.G. (1984) Perforation of the gallbladder. *Acta Chir. Scand.*, **150**, 557–560
4. Van Der Linden, W. and Edlund, G. (1981) Early versus delayed cholecystectomy: the effect of a change in management. *Br. J. Surg.*, **68**, 753–757
5. Keighley, M.R.B. (1982) Infection and the biliary tree. In: *Clinical Surgery International*, The Biliary Tract, pp. 219–235. Edited by L.H. Blumgart. London: Churchill Livingstone

Aws S. Salim, PhD,
Department of Surgery,
Ward 6,
Stobhill General Hospital,
Glasgow G21 3UW, UK

